# Pancreatic Tuberculosis Mimicking Pancreatic Tumor: A Case Report from Rural Area in Indonesia

**DOI:** 10.34172/mejdd.2024.379

**Published:** 2024-04-30

**Authors:** Agnestia Selviani Tanic, Laksmita Ayu Dewi Tetanel, Fransiskus Xaverius Rinaldi, Vania Levina Polanit, Ayers Gilberth Ivano Kalaij, Berti Julian Nelwan

**Affiliations:** ^1^Department of Internal Medicine, Karel Sadsuitubun Hospital, Maluku, Indonesia; ^2^Faculty of Medicine, Universitas Indonesia, Jakarta, Indonesia; ^3^Department of Anatomical Pathology, Faculty of Medicine, Universitas Hasanuddin, Makassar, Indonesia

**Keywords:** Pancreatic tuberculosis, Pancreatic tumor, Histopathology

## Abstract

Pancreatic tuberculosis (TB) is extremely rare and has similar clinical and radiological findings compared to pancreatic malignancy. Challenges in detecting individuals with pancreatic TB, especially in rural areas with limited supporting resources, are the reasons for a more complete care strategy. We report a case of pancreatic TB in a 25-year-old woman admitted to the emergency department (ED), who was initially suspected of having a pancreatic tumor. Her chief complaints were fever, fatigue, and abdominal pain, while she also experienced weight loss. Exploratory laparotomy and further pathological evaluation suggested pancreatic TB. Subsequently, the patient was given anti-TB drugs and showed clinical improvement. In conclusion, this case report highlighted that pancreatic TB could mimic pancreatic cancer; however, it is a treatable condition. Thus, it is important for physicians to consider this as a differential diagnosis, especially in high-risk populations and in rural areas with limited diagnostic tools.

## Introduction

 Tuberculosis (TB) remains one of the most common infectious diseases and a leading cause of mortality worldwide. In Indonesia, the annual TB incidence rate is 345 cases per 100 000 individuals, with a corresponding fatality rate of 52 cases per 100 000 individuals.^1^

 TBhas been known to be manifested in several regions of the body, including abdominal TB, with the incidence accounting for approximately 10% of extra-pulmonary TB.^2^ Pancreatic TB is an extremely rare abdominal TB that occurs in just 2.1%-4.7% of miliary TB autopsy findings and is typically found in immunocompromised persons.^3^

 The diagnosis of pancreatic TB remains a predicament and challenging situation, especially since it could mimic other diseases, typically due to the patient’s symptoms and clinical signs. Since a mass at the head of the pancreas can be observed on computed tomography (CT), it is frequently misleading to a pancreatic tumor diagnosis.^4^

 TB affecting the pancreas remains a clinical rarity, and the importance of the correct diagnosis is crucial to establishing a correct treatment and its prognostic implications, especially in rural areas with limited resources. This is the first case report of pancreatic TB mimicking pancreatic cancer in immunocompetent young women in Indonesia, especially in rural settings.

## Case Report

 A 25-year-old woman was presented to our emergency department (ED) on January 18, 2023 with a chief complaint of fever persisting for one week. The fever was intermittent for the past four months, which was alleviated by the administration of paracetamol. The patient also experienced epigastric pain that extended to the left abdomen for a duration of one week. The pain was accompanied by nausea, vomiting, and a decrease in appetite. The patient also experienced weight loss of approximately 5 kg during the previous month. The patient’s medical records did not indicate a prior diagnosis of TB. However, the patient had contact with their nephew, who was undergoing TB drug therapy around one year ago.

**Figure 1:**
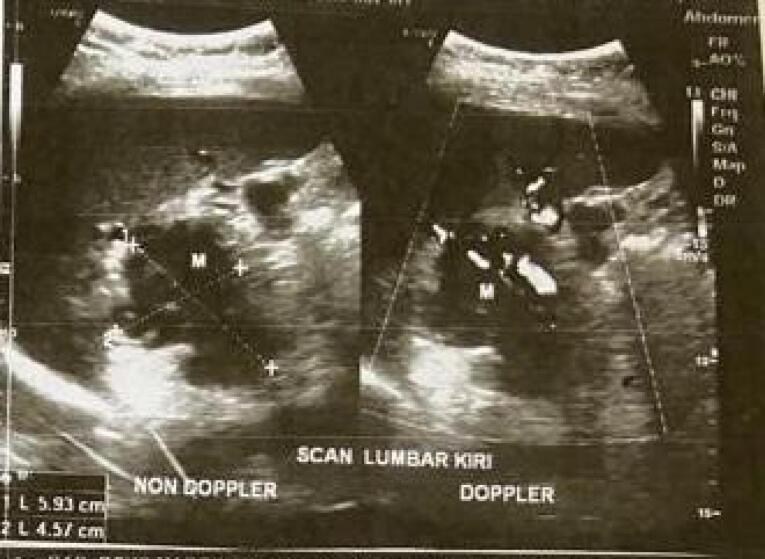
Abdominal ultrasound of the pancreas

 The physical examination revealed a temperature of 37.5 °C. The individual presented with pale conjunctiva and epigastric pain with visual analogue scale (VAS) 4. Other physical examination features were normal. The laboratory test revealed lymphocytopenia of 16% and neutrophilia of 80% with no abnormality in white blood cell count. The liver function was still within the normal level, the human immunodeficiency virus was not detected, and the GeneXpert test for TB was not detected. Chest radiograph showed no abnormality. The abdominal ultrasound scan revealed a well-defined mass with irregular edges, measuring 5.93 × 4.57cm, in the region between the spleen and the left kidney, suggestive of pancreatic mass (Figure 1). Subsequently, the patient was directed to a referral hospital possessing more adequate facilities for further evaluation.

 The patient underwent a CT scan, which revealed the presence of a cauda pancreatic solid-cystic mass measuring 12 cm × 10 cm. During the surgical procedure, the surgeon selectively excised a portion of the pancreatic tissue and thereafter submitted it for anatomical pathology examination. The anatomical pathology examination identified the presence of a lump in the stomach, which was the first tissue preparation for a pancreatic head tumor. The subsequent tissue of the curvatora minor lymph gland was also conducted. Macroscopic clinical diagnosis revealed a pancreatic tumor with two initial tissue fragments of 1 cm × 1 cm × 0.5 cm, with a brownish-white coloration and a spongy texture. The second tissue comprised a tissue with dimensions of 2.5 cm × 1.5 cm × 1 cm, enclosed by a white, sticky capsule.

**Figure 2:**
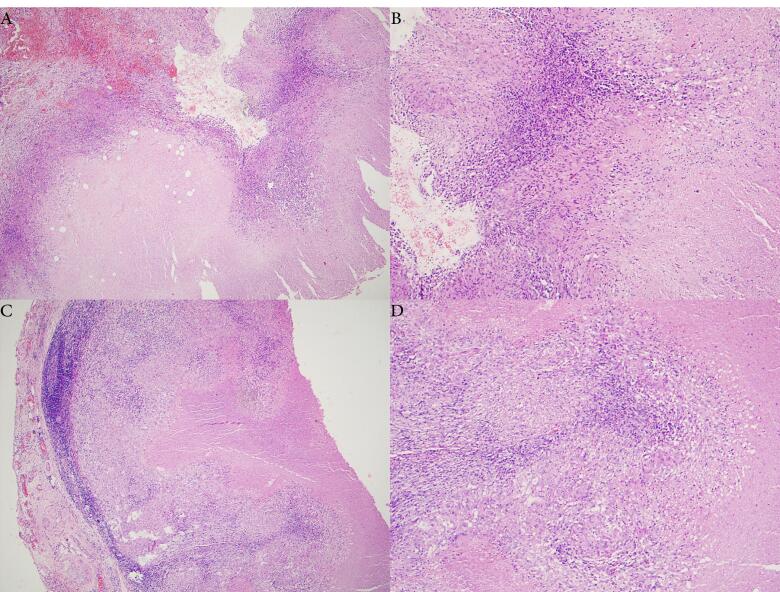
Microscopic evaluation of pancreatic tissue biopsy: (A) First slide with HE × 4;(B) First slide with HE × 10;(C) Second slide with HE × 4;(D) Second slide with HE × 10

 The microscopic analysis of the initial tissue sample revealed the lymphohistiocytic inflammatory cells, a fibrous matrix containing necrotic caseosa, Langerhans datia cells, and histiocytic epithelioid cells (Figures 2A and 2B). The second tissue sample exhibited lymphohistiocytic inflammatory cell clusters and areas of caseous necrosis. Langerhans datia cells and histiocytic epithelioid cells were observed between lymphoid follicles containing a lymphoid population (Figures 2C and 2D). Notably, no malignant characteristics were observed from this histopathology analysis, and it was concluded that both samples exhibited persistent granulomatous inflammation and granulomatous chronic lymphadenitis, respectively, which was attributed to a specific etiology of TB.

 The patient was provided with a presumptive diagnosis of pancreatic TB, and a treatment regimen consisting of isoniazid, rifampin, ethambutol, and pyrazinamide was prescribed for a duration of 9 months. Diagnosis of pancreatic TB was confirmed in a biopsy specimen culture two weeks following the surgical procedure. After a duration of 4 months following the initiation of the treatments, the patient had alleviation of abdominal pain and exhibited weight increase.

## Discussion

 The majority of pancreatic TB cases arise from contiguous infections originating from peripancreatic lymph nodes, with hematogenous spreading occurring only in rare cases. There have been three forms of pancreatic TB reported: (*a*) miliary TB, (*b*) spread from retroperitoneal lymph nodes to the pancreas, and (*c*) localized pancreatic TB.^3^

 The clinical symptoms might vary, including abdominal pain, weight loss, anorexia, jaundice, possibly fever, night sweats, palpable epigastric mass, and peripheral lymphadenopathy.^5^ Our patient experienced nausea, vomiting, lack of appetite, weight loss, and abdominal pain that were typical of pancreatic TB symptoms. However, further examination is required to confirm the diagnosis due to symptoms similar to cases of lymphoma or malignancy.^6^

 Direct histological confirmation obtained from an excisional biopsy is the most effective method for pancreatic TB diagnosis. Laparotomy for direct histological investigation should be limited to situations when imaging techniques and fine-needle aspiration cytology (FNAC) have failed to confirm a diagnosis. Typically, a biopsy is obtained from a pancreatic lesion and/or a lymph node near the pancreas.^4^ The ultrasonography and CT scan of the abdomen in our case report revealed a mass in the pancreas; therefore, the first diagnosis was a pancreatic tumor. A histological investigation of the tissue biopsy was done to corroborate the imaging results, and the appearance of necrotic caseosa was obtained. Caseous necrosis is a histological feature present in TB lesions. Epithelioid cells, lymphocytes, other mononuclear cells, and Langerhans giant cells can also be seen in this histological feature.^7^

 Upon the establishment of a diagnosis of pancreatic TB, most patients receive anti-tubercular pharmacological therapy, with surgical intervention being necessary in certain cases. Regardless, the outcomes of treatment were deemed satisfactory, as pancreatic TB led to death in only a limited number of cases. This fact further emphasizes the importance of a rapid diagnosis of pancreatic TB, as efficient treatment options are available even for immunocompromised patients.^8^

## Conclusion

 The predicaments and challenges in detecting individuals with pancreatic TB are the underlying reasons for further examinations and complete care strategies. This case is reported for the first time concerning pancreatic TB mimicking pancreatic cancer in an immunocompetent young Indonesian woman in a rural setting. It is important to raise the awareness of healthcare providers of the potential challenges in diagnosing pancreatic TB and in adding this condition to the differential diagnoses for patients with suspected pancreatic tumor.
